# Incident Reporting Behaviours and Associated Factors among Nurses Working in Gondar University Comprehensive Specialized Hospital, Northwest Ethiopia

**DOI:** 10.1155/2016/6748301

**Published:** 2016-12-28

**Authors:** Eshetu Haileselassie Engeda

**Affiliations:** School of Nursing, College of Medicine and Health Sciences, University of Gondar, Gondar, Ethiopia

## Abstract

*Background*. A comprehensive and systematic approach to incident reporting would help learn from errors and adverse events within a healthcare facility.* Objective*. The aim of the study was to assess incident reporting behaviours and associated factors among nurses.* Methods*. An institution-based cross-sectional study was conducted from April 14 to 29, 2015. Simple random sampling technique was used to select the study participants. Data were coded, entered into Epi Info 7, and exported to SPSS version 20 software for analysis. A multivariate logistic regression model was fitted and adjusted odds ratio with 95% confidence interval was used to determine the strength of association.* Results*. The proportion of nurses who reported incidents was 25.4%. Training on incident reporting (Adjusted Odds Ratio (AOR) [95% CI] 2.96 [1.34–6.26]), reason to report (to help patient) (AOR [95% CI] 3.08 [1.70–5.59]), fear of administrative sanctions (AOR [95% CI] 0.27 [0.12–0.58]), fear of legal penalty (AOR [95% CI] 0.09 [0.03–0.21]), and fear of loss of prestige among colleagues (AOR [95% CI] 0.25 [0.12–0.53]) were significantly associated factors with the incident reporting behaviour of nurses.* Conclusion and Recommendation*. The proportion of nurses who reported incidents was very low. Establishing a system which promotes incident reporting is vital.

## 1. Background

Developing a patient safety culture in a healthcare facility is among the priority recommendations by the international health community and incident reporting has been considered as an indispensable pillar [1, 2]. The first step in developing a patient safety culture is assessing the existing safety culture [3]. As revealed in a substantial number of literatures, common understanding on the importance of patient safety, communication based on mutual trust, synchronized information flow, commitment from the leaders, and the existence of nonpunitive approach for incident reporting were among the major predictors of positive patient safety culture in health organizations [3–5].

According to the literature, errors in the healthcare delivery system are inevitable, multidimensional, and everlasting threats to patient safety [6]. A diverse interaction of human behaviour, sociocultural features, technical aspects of a system, and a wide range of system weaknesses contribute to medical errors [7]. Although all errors do not cause harm to patients, professional errors exhibit system's vulnerability [6].

An inclusive and systematic approach to incident reporting would help learning from errors and adverse events within the same setup [8]. Moreover, the record would serve as a source of learning that helps other patients in different parts of the world [7]. Through incident reporting, various kinds of errors can be traced and discussed among health professionals and preventive mechanisms can be designed [6].

Incident reporting is a mechanism which enables health professionals to disclose unintended injury and near misses caused by a healthcare system or a health professional [9].

Despite the significant contribution of incident reporting to patient safety, the magnitude of underreporting remains high in different countries across the globe [10]. For instance, it occurs at a rate of 50%–96% in the United States [11]. Studies in the United Kingdom demonstrated that despite having awareness about incident reporting, 25% of health professionals do not know how to access incident reporting form and more than 40% of consultants and registrars had never completed a report [2].

Several factors were found to be associated with nonreporting or underreporting of incidents. Participants of a qualitative study in Korea suggested ninety-six barriers to incident reporting in their hospitals. These barriers were categorized into individual and organizational levels. Some of the most frequently reported barriers include the following: low rates of reporting, poorly designed incident reporting systems, and lack of adequate patient safety leadership [12]. A year before, a similar study among Iranian nurses revealed barriers associated with nurses' perceptions which include the following: fear of legal action and job threats, fear of economic losses, and fear of honour and dignity [6].

Among the interventions so far implemented to improve incident reporting are training on incident reporting, reducing fear of reporting, reducing reporting burden, and improving feedback system [9]. A Dutch study which evaluated local and central incident reporting systems suggested that a local incident reporting procedure motivates health professionals to report. Unlike local reporting systems, as described in the same study, the central incident reporting system was found to be useful in addressing generic and recurring patient safety concerns [13].

In Ethiopia, the Ministry of Health has designed strategies, procedures, and processes for patient care quality which included an incident reporting system. According to the Ethiopian hospital reform implementation guideline, an incident officer should be assigned to each hospital to receive and investigate all incident reports. The officer should investigate all reports received (ideally with the team leader of the ward where the incident came from) and should determine whether further action is necessary. A summary report of all incidents must be submitted to a quality assurance committee of each hospital. Each hospital must also report to the Ministry and the Food, Medicines, and Health Care Administration agency (a governmental regulatory agency in Ethiopia) [14].

Although quite a lot of studies are available regarding incident reporting mainly in the western countries, very limited information exists in Ethiopia particularly in the study area. Therefore, this study is aimed at assessing incident reporting behaviours and associated factors among nurses in order to help the hospital design appropriate incident reporting mechanism.

## 2. Materials and Methods 

Institution-based cross-sectional study was conducted from April 14 to 29, 2015 in Gondar University Comprehensive Specialized Hospital. The hospital is found in Gondar town 748 km far from Addis Ababa to the Northwest of Ethiopia. It is a teaching hospital which acts as a referral center for the nearby general hospitals. Having more than 500 inpatient beds, it provides referral services for over 5 million inhabitants in the Northwest region of Ethiopia.

The sampling frame for this study was employed nurses working in Gondar University Comprehensive Specialized Hospital. Sample size was determined using single population proportion formula by considering the following assumptions: 95% confidence interval (CI), 50% proportion (since there was no previous study), and 5% marginal error. By adding 10% nonresponse rate, the final sample size was 423. Simple random sampling technique was used to select the study participants.

The outcome measure of this study was incident reporting behaviour (yes/no) measured with a three-item scale (“when a mistake is made, but is caught and corrected before affecting the patient, how often is this reported?”, “when a mistake is made but has no potential harm to the patient, how often is this reported?”, and “when a mistake is made, that could harm the patient, but does not, how often is this reported?”). The items were answered on a five-point Likert scale with response options ranging from 1 (not reported at all) to 5 (always reported). The independent variables included sociodemographic characteristics (age, sex, nursing educational level, years of service as a nurse, working hours per week, and primary work area), institutional factors (training on incident reporting, availability of guideline/policy, and availability of reporting formats), and perceived barriers of incident reporting.

English version of structured and pretested self-administered questionnaire was used to collect the data (English is the media of instruction in all Ethiopian nursing schools).

Four diploma holder nurses (in the Ethiopian context a diploma nurse is the one who studied nursing for 3 years in a known nursing school) for data collection and two BSC holder nurses for supervision were recruited during the data collection period (both the data collectors and supervisors were not from the same hospitals). The aim of the study was clearly explained to the study participants before they filled the questionnaire. The data collectors and supervisors were trained separately for one day on how to facilitate the data collection process and prevent errors. Questionnaires were reviewed and checked for completeness, accuracy, and consistency by supervisors and the principal investigator every day during the data collection period.

The data were coded, entered into Epi Info 7, and exported to SPSS version 20 software for analysis. At the beginning of the analysis, summation of the scores for the scale incident reporting behaviour was made. Then the variable was recoded and dichotomized. Therefore, the Likert scale data were analyzed as categorical data (the scores for three outcome variables were summed up and then categorized). Descriptive statistics was used to illustrate the means, standard deviations, medians, and frequencies of the study variables. Bivariate analysis was computed and those variables whose *P* values are less than or equal to 0.2 were fitted into the backward stepwise multivariate logistic regression model. Odds ratios with 95% confidence interval were used to determine the strength of association between dependent and independent variables. Moreover, *P* values less than or equal to 0.05 were considered as statistically significant.

Ethical clearance was obtained from university of Gondar Institutional Review Board. The aim of the study was clearly explained to participants and hospital officials. The data collection was begun after obtaining consent from each participant. Confidentiality was maintained by excluding the names of participants from questionnaires. No other person except the data collection facilitators and the principal investigator had access to the filled questionnaires.

For the purpose of this study, an incident was defined as an injury, a medical error, and/or a near miss caused by a healthcare system or a health professional unintentionally.

## 3. Results

Of the total 423 nurses contacted to participate in the study, 378 returned the questionnaire for a response rate of 89.4%. Fifty-four percent of the participants were males. The mean (SD) age was 31.86 (9.65) years. Approximately 87% of the participants had a bachelor degree in nursing and nearly 44% of the participants served less than or equal to five years in the nursing profession. Most of the respondents (94.7%) were working as staff nurse. The mean (SD) working hours per week were 47.31 (10.59) hours (Table 1).

### 3.1. Incident Reporting Behaviours of the Participants

In this study, the proportion of nurses (after summation of all the three outcome variables) who reported incidents in all situations was 25.4%. The incident reporting behaviour of participants in different situations of patients is presented in Table 2.

### 3.2. Bivariate and Multivariate Findings: Incident Reporting Behaviour and Associated Factors

In the bivariate logistic regression analysis, age, sex, responsibility of the nurse, taking training on incident reporting, availability of reporting guideline, availability of reporting format, deciding if reporting is to help a patient, fear of administrative sanctions, fear of legal penalty, and fear of loss of prestige among colleagues met the criteria to be fitted into a multivariate analysis (*P* value less than or equal to 0.2) (Table 3). However, in the multivariate analysis, only training on incident reporting, reason to report (to help a patient), fear of administrative sanctions, fear of legal penalty, and fear of loss of prestige among colleagues were yielded as significantly associated factors with incident reporting behaviour of nurses.

Training was significantly associated with the incident reporting behaviour of nurses. Nurses who had received training on incident reporting were more likely to report incidents than those who had not been trained (AOR = 2.96, 95% CI: 1.34, 6.26). Those who believed reporting helps a patient were more likely to report incidents than those who did not believe so (AOR = 3.08, 95% CI: 1.70, 5.59). Nurses who feared administrative sanctions were less likely to report incidents than those who did not fear administrative sanctions (AOR = 0.27, 95% CI: 0.12, 0.58). Similarly, nurses who feared legal penalties were less likely to report incidents than those who did not fear legal penalties (AOR = 0.09, 95% CI: 0.03, 0.21). Moreover, nurses who feared loss of prestige were less likely to report incidents than those who did not fear loss of prestige (AOR = 0.25, 95% CI: 0.12, 0.53) (Table 4).

## 4. Discussion

The aim of this study was to assess incident reporting behaviour and associated factors among nurses. The study identified that training on incident reporting, reporting of incidents to help a patient, fear of administrative sanctions, fear of legal penalty, and fear of loss of prestige as significant predictors of nurses' incident reporting behaviour.

When health institutions are able to establish a successful incident reporting system, patient safety could be improved and the system would allow nurses and other clinicians to have easy access to reporting an incident with an understanding that their report could be handled in a nonpunitive manner [15]. Moreover, the reported incidents would be used in a positive way in that health professionals could learn from their mistakes and improve healthcare system and service without fear of administrative and/or legal consequences [16]. On top of that, such kind of system fosters enhanced learning regarding the causes of incidents and helps health institutions effectively design a sustainable mechanism to prevent incidents from recurring.

In this study, training was significantly associated with the incident reporting behaviour of nurses. Nurses who had received training on incident reporting were more likely to report incidents than those who did not receive training about incident reporting. This finding is in line with other similar studies in that training on incident reporting and patient safety issues will significantly improve the nurses' knowledge in sentinel events and near misses [17]. Moreover, it will also increase the nurses' skill in analyzing incidents in order to determine their causal factor so that they will be able to prevent future occurrences of similar events and obviously be motivated to report new incidents. On top of that, trained nurses will fully understand what constitutes patient safety and have the benefit of learning from mistakes [10, 12]. On the other hand, as supported by the literature, trained nurses will become open-minded, consider the benefit of error reporting, keep on self-training, engage in training of others, can obtain frequent feedback from others, and will accept incident reporting as a norm [6].

The present study showed that those nurses who believed that reporting could help patients were more likely to report incidents than those who did not believe so. This finding could be attributed to the fact that the perception of nurses on the benefit of reporting a particular incident plays an important role in whether to report it or not. In most cases, nurses would like to work for the benefit of patients by avoiding any sort of harm as much as possible. This will help nurses to be motivated in adverse event reporting as far as it is for the benefit of the patient despite limitations in understanding or knowledge of all kinds of harmful incidents. Studies also showed that reporting practices were attributed to staff views on the importance of the incident to the patient [18].

Consistent with other international studies [6, 19], the current study also showed that nurses who feared administrative sanctions were less likely to report incidents than those who did not fear administrative sanctions. This finding could be attributed to the fact that many nurses demonstrated perceived fear of organizational sanctions of variable degrees if they report incidents. According to the literature, perceived administrative measures include but are not limited to fear of economic sanction and fear of losing one's position. Moreover, many nurses also have a fear that their case could be examined in the medical or nursing council and disciplinary measures could be taken. The nurses might also fear that disciplinary measures could have huge impact on their future career. According to the local context, one's job profile plays a very crucial role for his/her future career. Therefore, nurses who perceived that incident or medical error reporting would be followed by oral or written warning, demotion, a delay in promotion, or dismissal from the organization could hold the incident from reporting unless there is a system that protects them from such kind of administrative interference.

In this study, nurses who feared legal penalties were less likely to report incidents than those who did not fear legal penalties. This could be attributed to a perceived severity of the incident. Basically, incidents or medical errors might be categorized into four categories. The first one could be an incident which happened but had been caught and corrected before affecting the patient. The second one could be an incident which happened and was recognized later but has no potential harm to the patient. The third one could be an incident, which could potentially harm the patient, which happened and was recognized later but fortunately did not harm the patient. Lastly, there could also be a kind of incident which happened and was recognized later after harming the patient. According to the literature, significant number of nurses reported that an incident report could be used against them and there would not be legal protection particularly if the incident has actual or potential adverse effect on the patient [6, 18]. Moreover, according to the Penal Code of Ethiopia, it is punitive to hurt a patient as a result of negligence or medical error even if it is unintentional. Therefore, the nurses may not report incidents because of fear of legal consequence.

In line with other international studies [12, 19], this study also showed that nurses who feared loss of prestige were less likely to report incidents than those who did not fear loss of prestige. The possible explanation for the current finding could be attributed to the fact that if nurses report incidents they perceive that they could be considered as incompetent among their colleagues and lose their prestige.

One of the possible limitations of this study could be its cross-sectional nature in which it does not confirm definitive cause and effect relationships between the outcome variable and associated factors. On the other hand, since self-reported data had been used and the questionnaire was not validated in the local context, the reliability and validity of the results might be negatively influenced to some extent.

Although factors associated with incident reporting behaviour of nurses have been identified in this study, further large scale study is required (if possible triangulated with qualitative design) to assess the situation across the country.

## 5. Conclusion 

This study showed that the overall proportion of nurses who reported incidents was very low. Better incident reporting behaviour was demonstrated when the incident had no potential harm to the patient. Training on incident reporting, reason to report (to help a patient), fear of administrative sanctions, fear of legal penalty, and fear of loss of prestige among colleagues were the factors significantly associated with the incident reporting behaviour of nurses. It is recommended that the hospital needs to train its nurses about the benefits of incident reporting with regard to ensuring and promoting patient safety. Moreover, it is strongly advisable to work on establishing a system which encourages nurses to report incidents in a way that they are protected from administrative sanctions and legal penalties.

## Figures and Tables

**Table 1:** Sociodemographic characteristics of nurses working in Gondar University Comprehensive Specialized Hospital, 2015 (*n* = 378).

Variables	Frequency	Percentage
Age category		
20–29	182	48.1
30–39	94	24.9
40–49	68	18
50–59	34	9
Sex		
Male	204	54
Female	174	46
Nursing educational level		
Master's degree	14	3.7
BSC degree	328	86.8
Diploma^*∗*^	36	9.5
Years of service as a nurse		
0–5 years	168	44.4
6–10 years	131	34.7
11–15 years	51	13.5
21 years and above	28	7.4
Responsibility of the nurse		
Team leader/coordinator	20	5.3
Staff nurse	358	94.7
Working hours per week		
21–39 hours	152	40.2
40–59 hours	177	46.8
60 hours and above	49	13
Primary area of work		
Medicine	86	22.8
Surgery	53	14
ICU	26	6.9
Gynaecology/obstetrics	26	6.9
Pediatrics	53	14
Neonate	20	5.3
Outpatient department	33	8.7
Emergency	50	13.2
Others^*∗∗*^	31	8.2

^*∗*^In the Ethiopian context, a diploma nurse is the one who studied nursing for 3 years in a known nursing college. A 4-year study is required for BSC degree.

^*∗∗*^Others include ophthalmic, fistula, and cancer treatment centre.

**Table 2:** Incident reporting behaviours in different patient situations among nurses working in Gondar University Comprehensive Specialized Hospital, 2015 (*n* = 378).

Situation	Frequency (%)	
	Reported	Did not report
Situation 1^*∗*^	41.5	58.8
Situation 2^*∗∗*^	26.2	73.8
Situation 3^*∗∗∗*^	37.3	62.7

^*∗*^When a mistake is made but is caught and corrected before affecting the patient, how often is this reported?

^*∗∗*^When a mistake is made but has no potential harm to the patient, how often is this reported?

^*∗∗∗*^When a mistake is made that could harm the patient but does not, how often is this reported?

**Table 3:** Bivariate analysis of factors associated with incident reporting among nurses working in Gondar University Comprehensive Specialized Hospital, 2015 (*n* = 378).

Variable	Incident reporting^*∗*^		Odds ratio (95% confidence interval) and *P* value	
	Yes	No	Crude odds ratio	*P* value
*Sociodemographic factors *				
Age category				
20–29	42	40	1	
30–39	28	66	1.41 (0.81–2.48)	0.20
40–49	23	45	1.70 (0.93–3.13)	0.09
50–59	3	31	0.32 (0.09–1.11)	0.07
Sex				
Male	46	158	1	
Female	50	124	1.39 (0.87–2.20)	0.16
Responsibility of the nurse				
Team leader/coordinator	9	11	1	
Staff nurse	87	271	0.39 (0.16–0.98)	0.05
*Organizational factors *				
Training on incident reporting				
Trained	21	39	1.75 (0.97–3.14)	0.07
Not trained	75	243	1	
Availability of guideline				
Yes	21	26	2.76 (1.47–5.18)	0.002
No	75	256	1	
Availability of reporting format				
Yes	18	32	1.80 (0.96–3.39)	0.06
No	78	250	1	
*Reason to report *				
To help the patient				
Yes	73	131	3.66 (2.17–6.18)	0.000
No	23	151	1	
*Barriers to incident reporting *				
Fear of administrative sanctions				
Yes	9	82	0.25 (0.12–0.53)	0.000
No	87	200	1	
Fear of legal penalties				
Yes	6	76	0.18 (0.08–0.43)	0.000
No	90	206	1	
Fear of loss of prestige among colleagues				
Yes	12	82	0.35 (0.18–0.67)	0.002
No	84	200	1	

^*∗*^After summing up the scores of the three outcome variables.

**Table 4:** Multivariate analysis of factors associated with incident reporting among nurses working in Gondar University Comprehensive Specialized Hospital, 2015 (*n* = 378).

Variable	Incident reporting^*∗∗*^		Odds ratio (95% confidence interval)	
	Yes	No	Crude	Adjusted
*Sociodemographic factors *				
Age category				
20–29	42	40	1	
30–39	28	66	1.41 (0.81–2.48)	0.96 (0.49–1.88)
40–49	23	45	1.70 (0.93–3.13)	1.53 (0.73–1.24)
50–59	3	31	0.32 (0.09–1.11)	0.41 (0.10–1.58)
Sex				
Male	46	158	1	
Female	50	124	1.39 (0.87–2.20)	0.98 (0.55–1.78)
Responsibility of the nurse				
Team leader/coordinator	9	11	1	
Staff nurse	87	271	0.39 (0.16–0.98)	0.39 (0.12–1.27)
*Organizational factors *				
Training on incident reporting				
Trained	21	39	1.75 (0.97–3.14)	**2.96 (1.34–6.26)**
Not trained	75	243	1	
Availability of guideline				
Yes	21	26	2.76 (1.47–5.18)	1.98 (0.74–5.26)
No	75	256	1	
Availability of reporting format				
Yes	18	32	1.80 (0.96–3.39)	1.33 (0.53–3.29)
No	78	250	1	
*Reason to report *				
To help the patient				
Yes	73	131	3.66 (2.17–6.18)	**3.08 (1.70–5.59)**
No	23	151	1	
*Barriers to incident reporting *				
Fear of administrative sanctions				
Yes	9	82	0.25 (0.12–0.53)	**0.27 (0.12–0.58)**
No	87	200	1	
Fear of legal penalties				
Yes	6	76	0.18 (0.08–0.43)	**0.09 (0.03–0.21)**
No	90	206	1	
Fear of loss of prestige among colleagues				
Yes	12	82	0.35 (0.18–0.67)	**0.25 (0.12–0.53)**
No	84	200	1	

^*∗∗*^After summing up the scores of the three outcome variables.
